# *AtCIPK16*, a CBL-interacting protein kinase gene, confers salinity tolerance in transgenic wheat

**DOI:** 10.3389/fpls.2023.1127311

**Published:** 2023-03-16

**Authors:** Khadija Imtiaz, Moddassir Ahmed, Nazish Annum, Mark Tester, Nasir A. Saeed

**Affiliations:** ^1^ Wheat Biotechnology Lab, Agriculture Biotechnology Division, National Institute for Biotechnology and Genetic Engineering, Constituent College Pakistan Institute of Engineering and Applied Sciences, Faisalabad, Pakistan; ^2^ Center for Desert Agriculture, King Abdullah University of Science and Technology, Thuwal, Saudi Arabia

**Keywords:** CBL-CIPK, CIPK16, K^+^ homeostasis, MIFE, salt tolerance, Wheat

## Abstract

Globally, wheat is the major source of staple food, protein, and basic calories for most of the human population. Strategies must be adopted for sustainable wheat crop production to fill the ever-increasing food demand. Salinity is one of the major abiotic stresses involved in plant growth retardation and grain yield reduction. In plants, calcineurin-B-like proteins form a complicated network with the target kinase CBL-interacting protein kinases (CIPKs) in response to intracellular calcium signaling as a consequence of abiotic stresses. The *AtCIPK16* gene has been identified in *Arabidopsis thaliana* and found to be significantly upregulated under salinity stress. In this study, the *AtCIPK16* gene was cloned in two different plant expression vectors, i.e., *pTOOL37* having a *UBI1* promoter and *pMDC32* having a *2XCaMV35S* constitutive promoter transformed through the *Agrobacterium*-mediated transformation protocol, in the local wheat cultivar *Faisalabad-2008*. Based on their ability to tolerate different levels of salt stress (0, 50, 100, and 200 mM), the transgenic wheat lines *OE1*, *OE2*, and *OE3* expressing *AtCIPK16* under the *UBI1* promoter and *OE5*, *OE6*, and *OE7* expressing the same gene under the *2XCaMV35S* promoter performed better at 100 mM of salinity stress as compared with the wild type. The *AtCIPK16* overexpressing transgenic wheat lines were further investigated for their K^+^ retention ability in root tissues by utilizing the microelectrode ion flux estimation technique. It has been demonstrated that after 10 min of 100 mM NaCl application, more K^+^ ions were retained in the *AtCIPK16* overexpressing transgenic wheat lines than in the wild type. Moreover, it could be concluded that *AtCIPK16* functions as a positive elicitor in sequestering Na^+^ ions into the cell vacuole and retaining more cellular K^+^ under salt stress to maintain ionic homeostasis.

## Introduction

Agricultural sustainability depends upon growing suitable crops in a particular ecological environment to achieve optimum crop productivity (Annum et al., 2022). Abiotic stresses such as salinity, drought, heat, frost, nutrient, and metal toxicity are the major factors affecting grain quality and limiting crop yield. Salinity is one of the major abiotic stresses that adversely affect agricultural productivity and sustainability, therefore threatening food security at the national and global scales (Syed et al., 2021).

Salinity affects plant growth and survival through osmotic or ionic stresses resulting in higher accumulation of sodium and chloride ions in the plant cells (Salim and Raza, 2020; Pastuszak et al., 2022). Excessive accumulation of Na^+^ results in poor aeration, reduced infiltration, and reduced hydraulic conductivity (water movement) through soil/water (Hailu and Mehari, 2021). Out of the 14 billion hectares of available land on earth, 831 million hectares are salt-affected, which accounts for 6% of the total available land (Ding et al., 2023). Out of the 831 million hectares of salt-affected land, 434 million hectares are affected by sodic soil and the remaining 397 million hectares are categorized as saline soil. In Pakistan, 4.5 million hectares of agricultural land are affected by salinity (Farid et al., 2018; Syed et al., 2021).

Over the years, extensive research has been carried out to understand the cellular and molecular mechanisms responsible for salt tolerance in plants. The molecular mechanisms associated with salt tolerance in plants are classified as transporters, detoxifiers, osmotic adjusters, phytohormones, and salt sensors. Transporters increase sodium/chloride ion efflux or compartmentalize into the vacuole and balance sodium/potassium homeostasis through potassium channel 1 (AKT1), potassium transporter 5 (HAK5), S-type anion channel (SLAH1), and salt overly sensitive 1 (SOS1). Detoxifiers that scavenge ROS are produced by ascorbate peroxidase, superoxide dismutase, catalase (CAT), GPX, GSH/glutathione, and NADH:peroxiredoxin oxidoreductase enzymes. Osmotic adjusters including glycine, betaine, polyphenol, sugars, proline, free amino acids, and polyamines have the ability to maintain low intracellular osmotic potential under salt stress in plants. Phytohormones like abscisic acid, cytokinin, gibberellic acid, brassinosteroids, jasmonates, indole acetic acid, and ethylene facilitate a broad array of adaptive responses and signaling in response to salt stress (Shabala et al., 2015; Amarasinghe et al., 2020.

Salt stress creates depolarization of the plasma membrane due to Na^+^ entry into the cell and initiates the outward potassium ion (K^+^) current to repolarize the plasma membrane potential. After repolarization of the plasma membrane with activation of the H^+^ pump by the phosphorylation process, the calcium ion (Ca^2+^) homeostasis is restored and potassium ion (K^+^) efflux channels are closed (Boudsocq and Sheen, 2013; Van Kleeff et al., 2018). The activation of the membrane and the restoration of its membrane potential (by maintaining inside negative potential) allowed in the potassium ions (K^+^) through the inward rectifying cation channel (IRCC) passive diffusion possibly through the H^+^/K^+^ symporter coupled with the downhill movement of H^+^ ions (Assaha et al., 2017).

Plants have established specific molecular and biochemical and physiological mechanisms to overcome the negative effect of salinity (El Sabagh et al., 2021). Plasma membranes play an important role in the transport of various ions/nutrients and vesicle transport across the membrane through host-specific proteins and are involved in downstream signal transduction pathways (Annum et al., 2022). In plants, calcium-facilitated signal transduction plays a regulatory role under biotic and abiotic stresses. Under salt stress, the SOS pathways are reported to be involved in the CBL–CIPK interaction to regulate the cellular ionic balance in plants. The salt overly sensitive pathway encoded a plasma membrane Na^+^/H^+^ antiporter that regulates the transport of Na^+^ ions from the cytoplasm and channels the sodium transport at a long distance from the plant root to the shoot. It also maintains the concentration of Na^+^ ions to a non-toxic level to increase salinity tolerance in plants (Ding et al., 2023).

To develop salt tolerance in plants, multiple transgenic approaches have been used by transforming salt tolerance genes through the *Agrobacterium*-mediated method in proliferating plant callus (Ishida et al., 2015), *in-planta* transformation protocol (Singh and Kumar, 2022), and chloroplast/plastid transformation through biolistic bombardment (Hanson et al., 2013). Several salt-tolerant genes isolated from various genetic resources have been transformed into cereal and vegetable crops to confer salinity tolerance. Overexpression of the *NHX1* gene from *Arabidopsis thaliana* has demonstrated salinity tolerance in tomato, the *AtAVP1* gene is associated with improving salinity tolerance in barley, the *TaHKT*1 gene excluded Na^+^ ions from the cell to develop salinity tolerance in durum wheat, and the expression of *AtCIPK16* proved to improve salt tolerance in barley (Roy et al., 2013). In plants, four main Ca^+2^ ion sensors, namely, calmodulin (CaM), calmodulin-like proteins (CMLs), calmodulin-dependent protein kinase (CDPK), and calcineurin-B-like proteins (CBLs), receive the signals and temper the response due to rise in Ca^+2^ levels of different stimuli. In response to stress, CBLs as exclusive unique Ca^+2^ receptors sense the changes and bind with the CIPKs to develop a CBL–CIPK complex for downstream phosphorylation with the target protein to improve the plant stress-response ability (Xiaolin et al., 2022). The CBL–CIPK signaling is explicitly reported in plants (*Arabidopsis*, cotton, rice, etc.) as a Ca^2+^ ion-dependent system known to facilitate abiotic stress tolerance and is also involved in plant growth and development (Xiaolin et al., 2022). Therefore, the complexity and variety of CBL–CIPK signaling systems and the spatial specificity of target recognition were determined by their interacting function. CBLs were found to be two- to four-fold less in numbers as compared with CIPKs in green plants. The salt-responsive CBL–CIPK signaling mechanism exhibited much complexity in the functional diversity and expression pattern regulation due to diversity in the interaction of the different Ca^2+^ sensors with the same kinases and downstream phosphorylation of different target proteins (Tong et al., 2021).

In *A. thaliana*, abiotic stresses such as salinity, cold, and drought stimulate the CBL–CIPK gene expression (Tong et al., 2021). Recently, CBL–CIPK expression contributing to salinity tolerance had been verified in *A. thaliana* and *Oryza sativa* (Xi et al., 2017; Li et al., 2019). A sum of 10 loci encoding CBLs and 26 CIPKs encoding loci was found in *A. thaliana*, 10 CBLs and 34 CIPKs were determined in rice (*O. sativa*), 9 CBLs and 14 CIPKs were reported in barley (*Hordeum vulgar*e), 7 CBLs and 13 CIPKs were demonstrated in soybean (*Glycine max*), and 2 CBLs and 7 CIPKs were identified in pine (*Pinus*) (Ma et al., 2020; Tong et al., 2021; Xiaolin et al., 2022). *Arabidopsis thaliana* CIPKs (*CIPK1*, *CIPK3*, *CIPK6*, *CIPK8*, *CIPK16*, *CIPK21*, *CIPK23*, and *CIPK24*) had been known to be involved in salinity tolerance in plants (Amarasinghe et al., 2020; Ding et al., 2022). In the wheat genome, approximately 24 CBL and 79 CIPK genes had been identified (Ma et al., 2020). However, the function and related molecular mechanism of the CIPK gene family associated with abiotic stress tolerance have yet to be explored, especially in wheat. The *CIPK29* gene was identified in wheat and found to have a higher transcription level upon exposure to sodium, cold, ethylene, and methyl viologen. Salt stress changes the cytosolic calcium level in the root cells; these changes had been sensed and responded to the sensor molecules. Sensors (CBLs/Ca^2+^-binding domains) phosphorylate *AtCIPK16* to relieve it from autoinhibition to further phosphorylate multiple downstream targets. In *A. thaliana*, *AtCIPK16* was reported to possess a strong affinity with CBL4 and CBL5 rather than with CBL1, CBL2, CBL9, and CBL10 (Amarasinghe et al., 2020). *AtCIPK16* has also been constitutively overexpressed in a barley cultivar (Golden promise) and reported to improve salinity tolerance with a 20%-45% increase in plant biomass compared with traditional breeding. *ZmCIPK16* was identified in maize as a candidate gene to develop salinity tolerance in this crop plant species (Zhao et al., 2009; Ding et al., 2023).

A growing number of evidence explaining the role of *AtCIPK16* in salinity tolerance were investigated in model systems, and it was found to be highly conserved in many plant species through evolutionary descent but had yet to be evaluated in crop plants like wheat (Xiaolin et al., 2022). Therefore, the present study focused on the transformation of the *AtCIPK16* gene in a local wheat cultivar to investigate its involvement in imparting salinity tolerance in this crop plant.

## Materials and methods

### Plant material

Seeds of the local wheat cultivar *Faisalabad-2008* were obtained from the Wheat Research Institute (WRI), Ayub Agriculture Research Institute (AARI), Faisalabad, Pakistan. The seeds were sown in pots and grown in a glasshouse at the National Institute for Biotechnology and Genetic Engineering (NIBGE), Faisalabad, under the following growth conditions: a photoperiod of 14/10 h (day/night), a temperature of 22°C with 70% humidity, and a light intensity of 116 μmol m^−2^ s^−1^. Standard agronomic practices were followed to maintain optimum plant growth in pots under control conditions. Two plasmid constructs, i.e., expressing the *AtCIPK16* gene under the *UBI1* promoter in the *pTOOL37* plasmid (construct 1) and possessing the same gene under the *2XCaMV35S* promoter in the *pMDC32* plasmid (construct 2), were prepared and transformed in the local wheat cultivar *Faisalabad-2008* through *Agrobacterium*-mediated wheat transformation protocol. Non-transformed *Faisalabad-2008* plants were used as wild-type (*Wt*) control. Six transgenic wheat lines of overexpressing (*OE*) *AtCIPK16* gene were selected to evaluate various salinity levels in this study. The transgenic lines *OE1*, *OE2*, and *OE3* were expressing the *AtCIPK16* gene under the *UBI1* promoter, while the lines *OE5*, *OE6*, and *OE7* were expressing the same gene under the *2XCaMV35S* promoter. Plants were grown in small pots containing peat moss in a greenhouse with a 16/8-h day length regime at 20°C, 70% humidity, and a light intensity of 116 μmol m^−2^ s^−1^. Leaf samples from the transgenic wheat lines were collected from the greenhouse and processed for further experimentation.

### Preparation of the *AtCIPK16* plasmid constructs

Total RNA was isolated from the tissues of *A. thaliana* by following the RNase TRIzol reagent (Thermo Fisher Scientific, USA, Cat. # 15596026) method (Rio et al., 2010). The full-length cDNA of the *AtCIPK16* (Accession # AY030304.1) gene was amplified using gene-specific primers. The polymerase chain reaction (PCR)-amplified product was purified using a PCR Purification Kit (Thermo Fisher Scientific, Cat. # K0701) and cloned under the *UBI1* and *2XCaMV35S* promoters. Restriction analysis was performed to confirm the successful integration of the *AtCIPK16* gene using the *Apa*1 and *Spe*1 restriction enzymes in the *pTOOL37* plasmid (construct 1) and the *Sal*1 and *Spe*1 restriction enzymes in the *pMDC32* plasmid (construct 2). The restricted plasmid and the *AtCIPK16* gene fragments were resolved on 1% agarose gel to validate the cloning of the said gene under the *UBI1* and *2XCaMV35S* promoters.

### Transformation of plasmids in *Escherichia coli*

The heat shock method was used to transform the said plasmids in the *Escherichia coli* strain *DH10α* competent cells at 42°C for 45 s. Plasmid DNA was isolated by using Miniprep Kit (GeneJET, Cat. # 0502) from the transformed *E. coli* broth culture at OD_600_ cell density. Eluted plasmids were quantified by using the NanoDrop (ND-2000, Thermo Scientific) spectrometer. Extracted plasmids and glycerol stocks of bacterial cultures were stored at −80°C to be used for *Agrobacterium tumefaciens* transformation.

### Transformation of *Agrobacterium tumefaciens*

A hypervirulent strain, i.e., *AGL-1* of *A. tumefaciens*, was transformed by both the verified plasmid constructs through the electroporation method. Electric shock was provided at 1.44 kV, capacitance of 20 µF, and resistance of 220 Ω. The successful transformants of both vectors in *A. tumefaciens* cells were confirmed by PCR. The *AGL-1* cells harboring the binary vectors *pTOOL37* and *pMDC32* were used for the inoculation of wheat immature embryos.

### Wheat transformation

Immature wheat embryos were isolated from 9-14 days-old post-anthesis spikes and cultured on the callus induction medium. One-week-old calli were inoculated with *A. tumefaciens* culture and shifted to the freshly prepared Murashige and Skoog (MS) basal medium supplemented with 400 mM of acetosyringone. Inoculated immature embryos (in Petri plates) were incubated in the dark at 28°C for 2 h, blot-dried, and cultured on MS basal medium containing 200 mg L^−1^ of Timentin and 200 mg L^−1^ of hygromycin for 2 weeks. After 15 days, the developing calli were subcultured on a freshly prepared callus induction medium.

### Growth and regeneration activity of the callus

The developing calli were kept on the subculturing MS basal medium supplemented with 200 mg L^−1^ of Timentin until the initiation of regeneration signs. Then, the calli were transferred to the freshly prepared IK4 (IAA and kinetin) medium containing 200 mg L^−1^ of hygromycin till the start of regeneration. The regenerated tiny plantlets were transferred to sterile jars having IK4 medium supplemented with 200 mg L^−1^ of hygromycin as a plant selection. After the emergence of the primary roots, the plantlets were shifted to small plastic pots filled with peat moss and compost in a 2:1 ratio. These plastic pots were tightly covered with air-tight polythene bags. Upon attaining sufficient growth of the plantlets, the polythene bags were removed from the top of the plastic pots, and the pots were supplied with the necessary fertilizers to meet the nutritional requirements of the wheat plants.

### Molecular analysis of the putative wheat transgenes

#### Antibiotic selection assay

Putative transgenic wheat plants were initially assessed and evaluated through antibiotic selection assay by two different methods: 1) antibiotic leaf pasting assay by cotton swab and 2) detached leaf dip assay in antibiotic-supplemented solution. For this purpose, the MS medium containing 200 mg L^−1^ of hygromycin was prepared and used for the leaf pasting assay. The surface of the fully developed fourth leaf from the bottom was washed with distilled water and dried, and the 2-3-cm area of the leaves was painted *in vivo* with antibiotic (200 mg L^−1^ of hygromycin) solution with a cotton swab. In the detached leaf dip assay, a 5-cm-long piece of the fourth leaf of the *Wt* and putative transgenic wheat plants was cut and immersed in media containing 200 mg L^−1^ of hygromycin antibiotic for a period of 15 days.

#### Validation of putative transgenic wheat plants through PCR

Putative transgenic wheat plants confirmed through antibiotic leaf pasting and leaf dip assay were further subjected to validation through PCR amplification. Wheat leaf samples were taken to isolate DNA by using the CTAB method (Richards et al., 1994) from the *Wt* and transgenic plants. Antibiotic-specific (plant selection), promoter-specific, promoter- plus gene-specific, and gene-specific primers were used to further confirm the transgenic and non-transgenic (wild-type) plants. The plasmid DNA of each construct was used as a positive control, and the non-transformed wheat cultivar *Faisalabad-2008* (wild type) was used as a negative control in the PCR reaction setup. Approximately 100 ng of genomic DNA from each sample was used as a template in PCR amplification. DreamTaq Green PCR Master Mix (Thermo Fisher Scientific, USA) was used in the PCR master mix reactions. The insertion of the binary vector *pTOOL37* containing the *AtCIPK16* gene under the control of the *UBI1* promoter (construct 1) was validated in putative transgenic wheat plants through PCR by using primer sequences given in **Table 1**. The PCR profile consisted of an initial denaturation at 95°C for 5 min, followed by denaturation at 95°C for 1 min, primer annealing at 58°C for 1 min, fragment extension at 72°C for 1 min (repeated 30 times), and final extension at 72°C for 10 min. The integration of the binary vector *pMDC32* expressing the *AtCIPK16* gene under the *2XCaMV35S* promoter (construct 2) in transgenic wheat plantlets was confirmed through PCR amplification in the same manner as discussed above. The primer sequences used for the confirmation of the transgene are given in **Table 1**. The PCR profile was set with an initial denaturation at 95°C for 5 min, denaturation at 95°C for 1 min, primer annealing at 54°C for 1 min, fragment extension at 72°C for 1 min, and final extension at 72˚C for 10 min. The PCR products were resolved on 1% agarose gel and visualized under UV light by using a gel documentation system (Bio-Rad, USA).

**Table 1:** List of primers.

Sr. No.	Gene	Forward primer (5′–3′)	Reverse primer (5′–3′)
**1**	*Hygromycin*	GGCGACCTCGTATATTGGGAAT	ACCGCAAGGAATCGGTCAAT
**2**	*UBI1-AtCIPK16*	GGAGAACACATGCACACTAAAA	CACGTTTGGATGACGGAGGA
**3**	*2XCaMV35S-AtCIPK16*	CAACCACGTCTTCAAAGCAA	CGTGGTTGGAACGTCTTCTT

#### Expression analysis through RT-PCR

The expression of the *AtCIPK16* (Accession # AY030304.1) gene was quantified in transgenic wheat plants by using the following primer pairs: forward primer, 5′CCACCGTGGTTTTCCTTAGA3′; reverse primer, 5′TCCGTCGTCTTTTTCTCGT3′. Total RNA was extracted by using the TRIzol reagent (Invitrogen, USA, Cat. # TR118) followed by DNase treatment to remove any possible genomic DNA contamination. The first-strand cDNA was synthesized using a cDNA synthesis Kit (Fermentas, USA, Cat. # K1631). The relative expression of the *AtCIPK16* gene was determined through comparative threshold cycle values. The *actin* gene (Accession # KC775782.1) with primer sequences, 5′CCGACCGTATGAGCAAGGAG3′ and 5′AAATTCGCCGTTACCTGCTG3′, was used for the normalization of the transcript level.

#### Assessment of salinity tolerance

Transgenic wheat plants expressing the *AtCIPK16* gene under the *2XCaMV35S* and *UBI1* promoters were evaluated for salinity tolerance at various levels of salt stress. Fifteen seeds of each transgenic line were grown on germination paper at room temperature to check their viability and rate of germination. Upon the start of germination, plantlets were transplanted into small plastic/PVC pots containing vermiculite. Four salinity treatments, i.e., 0, 50, 100, and 200 mM, were applied along with Hoagland nutrient solution at regular 1-week intervals to assess the level of salinity tolerance in different overexpressing transgenic wheat lines.

#### Ion flux measurement

Net K^+^ ion flux was measured by non-invasive microelectrode ion flux estimation (MIFE) as described by Zarza et al. (2019). Microelectrode was prepared by following the protocol of Wu et al. (2015) (**Supplementary S7**). For the determination of K^+^ ion efflux from the root cells, 5-day-old seedlings of transgenic wheat were immobilized in a Petri plate containing basic salt solution (BSM 0.2 mM, CaCl_2_ 5 mM, MES 0.5 mM, KCl 2 mM, Tris base, pH 6.0). The roots were separated from the shoot issues and immobilized horizontally for preincubation for 30 min in the salt solution (Zarza et al., 2019). Four seedlings were used randomly for each replicate. The reference electrode was prepared to be used as control, and the K^+^ ion microelectrode was calibrated to be positioned at a 40-µm distance from the root tissue surfaces in the root mature zone (Batistič and Kudla, 2009). Initially, the steady flux was measured for a period of 10 min, then 1 ml of 1,000 mM NaCl stock solution was added, and net K^+^ ion flux was recorded for 30 min. Three-dimensional micromanipulators of the inverted microscope were used with a magnification of ×200. The MIFE program interfaced with the CHART software was used for its calibration as used earlier for the K^+^ ion microelectrode. The travel range of the microelectrode was set to 70 µm between two positions, and the hydraulic manipulator controlled the movement of microelectrodes between designated positions.

#### Flux calculations

The MIFEFLUX software was used to calculate the K^+^ ion flux data generated from transgenic and *Wt* wheat root cells in controlled conditions and in NaCl-induced stress condition (Yun et al., 2018). Statistical analysis was conducted by using Microsoft Excel 2016 Windows 10 (RRID: SCR_016137, https://www.microsoft.com/en-gb/). The significance of data was predicted by exploiting one-way ANOVA and Student’s *t*-test (*P< 0.05). K^+^ ion flux values were plotted against designated time laps and changes in flux.

## Results

### Cloning of the *AtCIPK16* gene under the *UBI1* and *2XCaMV35S* promoters

**Figure 1:**
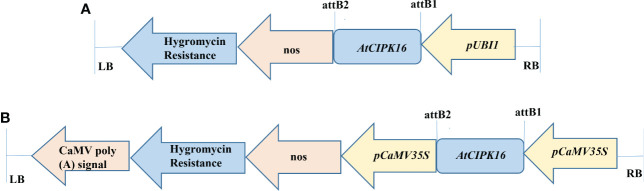
Diagrammatic representation of *AtCIPK16* constructs. **(A)** Construct 1 was used for wheat transformation (*pTOOL37* + *UBI1* + *AtCIPK16*) under the *Ubiquitin 1* promoter and the *pTOOL37* vector. **(B)** Construct 2 was used for wheat transformation (*pMDC32* + *2XCaMV35S* + *AtCIPK16*) under the *Cauliflower Mosaic Virus 35S* promoter and the *pMDC32* vector.

The *AtCIPK16* gene (1,410 bp fragment size) cloned in construct 1 and construct 2 (**Figures 1A, B**) was validated through restriction analysis of *pTooL37* and *pMDC32*, indicating the release of 2,131, 9,403, 2,154, and 10,131 bp cassettes (**Figures S1A, B**), respectively. These plasmid vectors containing the *AtCIPK16* gene were then transformed into the *AGL-1* strain of *A. tumefaciens*, and the transformants were confirmed by PCR amplification by plant selection (hygromycin)-specific (**Figure S2A**) and promoter- and gene-specific primers for construct 1 (**Figure S2B**) and promoter- and gene-specific primers for construct 2 (**Figure S2C** **;** **Table 1**).

### Selection of transformed wheat callus on antibiotic-supplemented medium

**Figure 2:**
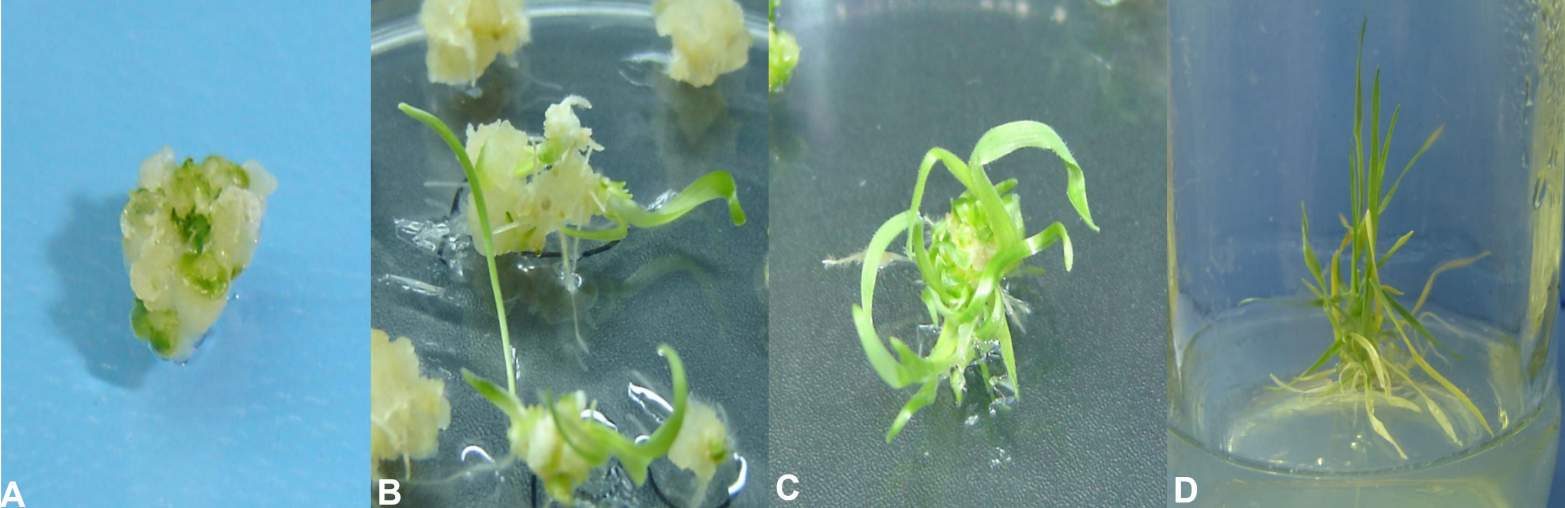
Stages of wheat tissue culturing and transformation. **(A)** Embryogenic callus. **(B)** Initiation of regeneration from embryogenic calli at IK4 regeneration media. **(C)** Profuse regeneration at the IK4 regeneration media. **(D)** Transfer of regenerated plantlets at the root induction media.

Upon transformation of the *AtCIPK16* gene into the wheat callus (**Figure 2**) by using the *Agrobacterium*-mediated protocol, 90 wheat plantlets originated from the inoculated calli grown on the selection medium. Among the 90 plantlets, only 28 were phenotypically identical to the parental wheat cultivar and started to have roots emerging on the rooting medium. Twelve lines were selected for further evaluation depending on their survival rate and potential to grow on the selection medium. No regeneration activity was observed in non-transformed wheat calli on the antibiotic selection medium.

### Antibiotic leaf pasting and leaf dip assay

**Figure 3:**
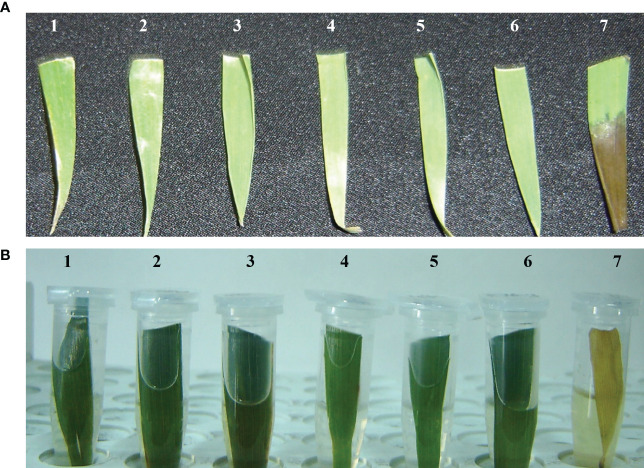
Antibiotic susceptibility assessment test comparing transformed wheat plants expressing *AtCIPK16* and non-transgenic wheat plants. **(A)** Leaf paint assay was performed by using 200 mg L^−1^ of hygromycin antibiotic selection; 1-6 are putative transgenic wheat plants, while 7 is a non-transgenic wheat plant. **(B)** Detached leaf dip assessment test of putative transgenic and non-transgenic wheat plants by applying 200 mg L^−1^ of hygromycin as antibiotic selection. Samples 1-6 are putative transgenic wheat plants, while 7 is a non-transgenic wheat plant.

**Figure 4:**
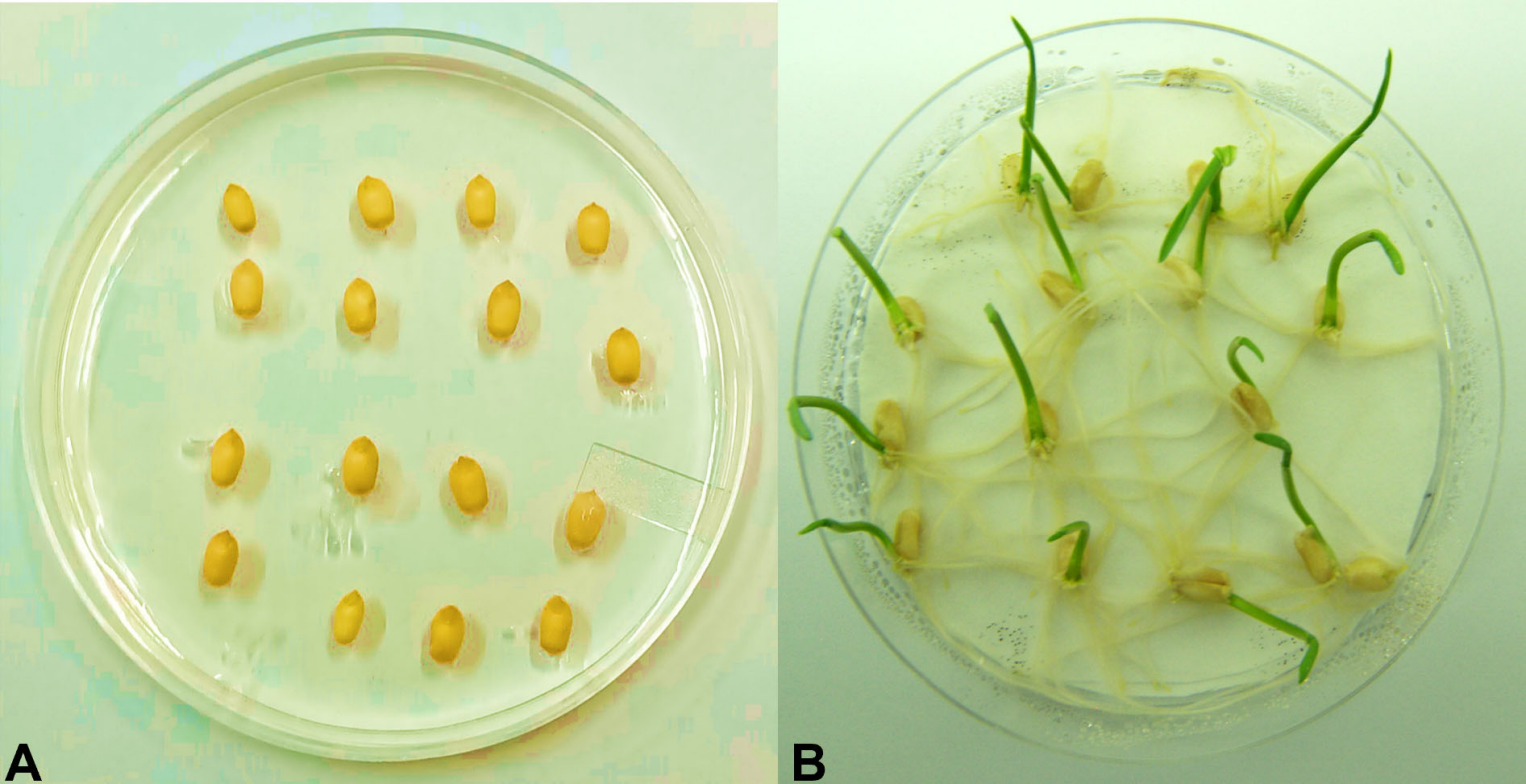
Germination assessment test. Wheat seeds of the T_1_ generation were grown on one-half strength MS media supplemented with 200 mM of NaCl and 200 mg L^−1^ of hygromycin antibiotic. **(A)** Non-transgenic (*Faisalabad-2008*) wheat plant. **(B)** Putative transgenic wheat plants expressing *AtCIPK16*.

Transgenic wheat plantlets surviving on the antibiotic selection medium were subjected to antibiotic leaf dip and leaf pasting assays. In the T_1_ generation of six transgenic wheat plants, their leaves stayed green in color 2 weeks after the application of 200 mg L^−1^ of hygromycin on leaf tissues. However, non-transgenic *Wt* showed tissue necrosis at the abovementioned dose of antibiotics. Necrotic lesions turned brown in color and became prominent in *Wt* plants (**Figure 3A**). The leaves of surviving plants were further verified by using the antibiotic leaf dip assay of both transgenic and non-transgenic plants in 200 mg L^−1^ of hygromycin solution. The leaves of transgenic wheat plants demonstrated less electrolyte leakage than the leaves of *Wt* control (**Figure 3B**). The seeds of the T_1_ generation were grown on MS medium supplemented with 200 mg L^−1^ of hygromycin and 200 mM of NaCl to induce salt stress in transgenic and non-transgenic wheat (**Figure 4**).

### Confirmation of *AtCIPK16* transgenic wheat by PCR and RT-PCR

**Figure 5:**
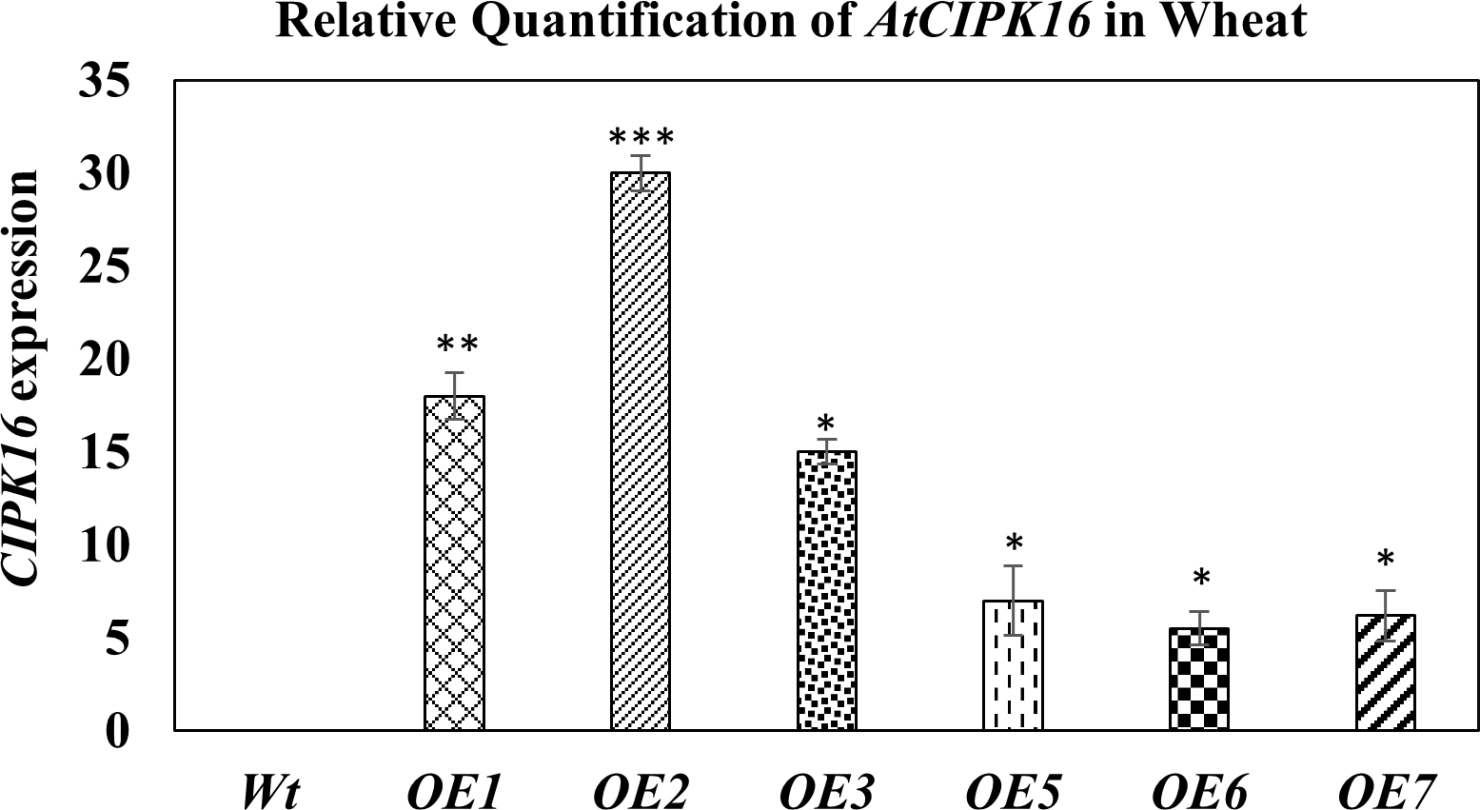
Expression analysis of the *AtCIPK16* gene in transformed wheat lines and wild-type control at 100 mM of NaCl concentration. *AtCIPK16* overexpressed lines showed a significant increase in expression profile in folds as compared to the control cultivar (*Faisalabad-2008*). Multiple comparison tests were performed to ascertain the significant difference between means at the significant level of *P* ≤0.05 (*), based on Student’s *t*-test, and represented as mean ± standard error (SE). The data presented are the means of four replicates.

Hexaploid wheat cv. *Faisalabad-2008* was transformed with the two constructs containing the *AtCIPK16* gene. Successful integration of these gene constructs in wheat plants was confirmed by PCR using gene-specific and promoter-specific (*UBI1* and *2XCaMV35S*) primers. PCR amplification of the *AtCIPK16* gene validated the integration of the transgene in six transgenic wheat lines which were selected earlier on antibiotic selection medium (**Figures S3, S4**). The relative expression of the *AtCIPK16* gene was performed in transgenic wheat lines by RT-PCR grown at 100 mM of NaCl, using *Wt* as negative control. The *AtCIPK16* transcript level was normalized by the *actin* gene. No increase in transcription level was observed in *Wt* plants of *Faisalabad-2008*. However, a significant increase was observed in the transcript level of the *AtCIPK16* gene in overexpressing wheat lines, i.e., *OE1*, *OE2*, *OE3*, *OE5*, *OE6*, and *OE7* (**Figure 5**). The transgenic lines *OE1*, *OE2*, and *OE3* showed 18×, 30×, and 15× higher expression, respectively, while the *OE5*, *OE6*, and *OE7* lines showed 7×, 5.5×, and 6.2× in transcript level as compared with the wild-type control.

### Assessment of transgenic wheat plants for salinity tolerance

**Figure 6:**
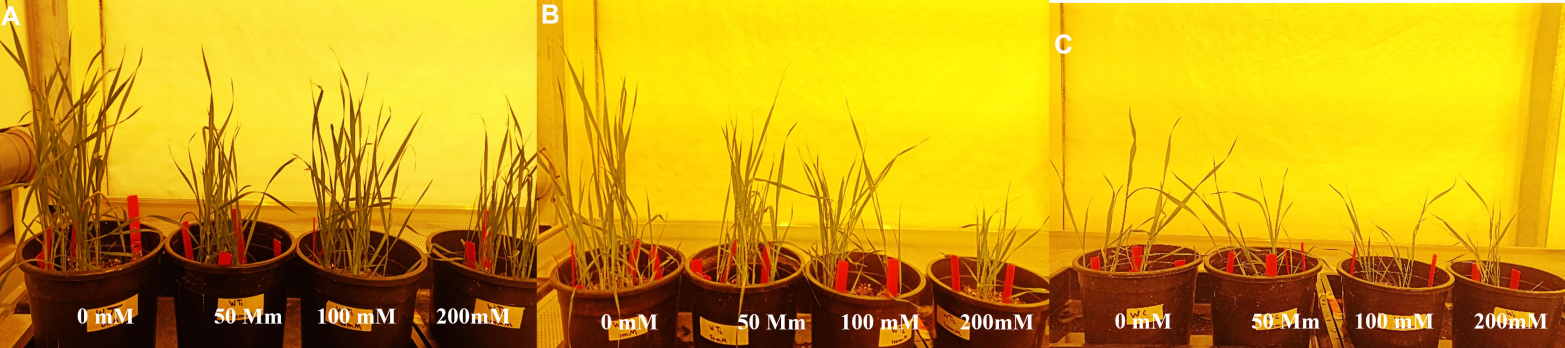
Assessment of transgenic wheat plants at 0, 50, 100, and 200 mM of NaCl concentrations. **(A)** Putative transgenic plants expressing gene cassette construct 1 (*pTOOL37* + *UBI1* + *AtCIPK16*) at different NaCl stress levels. **(B)** Putative transgenic plants expressing gene cassette construct 2 (*pMDC32* + *2XCaMV35S* + *AtCIPK16*) at different NaCl stress levels. **(C)** Seeds of wild type (*Wt*) (*Faisalabad-2008*) under different NaCl stress levels. Data were collected every week (2–4 weeks) to examine the germination rate and morphological parameters of plants under stress condition.

To determine the salinity tolerance level, *Wt* and wheat lines transformed with construct 1 and construct 2 were exposed to four NaCl treatments, i.e., 0, 50, 100, and 200 mM, under optimum growth conditions. Normal growth was observed in *Wt* and transgenic wheat lines at 0 mM of NaCl treatment. However, with the gradual increase in NaCl concentration, a significant reduction in plant growth/plant height and plant vigor was observed in both *Wt* and transgenic wheat lines (**Figure 6**). Interestingly, at 50 and 100 mM, the transgenic wheat plants produced more fertile tillers as compared with *Wt*. Additionally, at 200 mM of salt stress, *Wt* plants started dying. However, transgenic wheat lines survived upon exposure to 200 mM of NaCl treatment but produced some abortive tillers as well.

### NaCl-induced ion flux measurement in the root tissues of transgenic wheat

MIFE was used to determine NaCl-induced ion flux in root tissues of transgenic and *Wt* wheat (*Triticum aestivum* L.) plants. Under the saline condition, the excess NaCl was absorbed by the roots of the wheat plant, so the plasma membrane starts removing K^+^ ions from the cell to maintain cellular homeostasis at the beginning of salt stress. So, there was a need to establish a cellular mechanism which can help to retain K^+^ ions inside the cell to sustain normal cellular activity.

K^+^ ion fluxes along the length of the wheat root showed a variable pattern; in the mature zone of the wheat root, K^+^ ion fluxes were lower as compared with those in the elongation zone and root tip. When the microelectrode was positioned away from the elongation zone, a reduction in potassium efflux was observed. A sharp peak was observed in the elongation zone of the root that described a higher K^+^ ion efflux. Fluxes of different overexpressed wheat lines (*OE1*, *OE2*, *OE5*, *OE7*) were compared in the mature root zone, where the variability of ion fluxes is less than that in the elongation zone ((Wang et al., 2018). Four transgenic lines were selected based on their expression profile and level of salinity tolerance for MIFE analysis. The transgenic wheat lines *OE1* and *OE2* (overexpressing *AtCIPK16* under *UBI1*) and *OE5* and *OE7* (overexpressing *AtCIPK16* under *2XCaMV35S*) were selected to measure NaCl-induced ion flux in the root zone as compared with wild type. Five-day-old seedlings of these four *OE* lines (*OE1*, *OE2*, *OE5*, *OE7*) and *Wt* were exposed to 1 ml of 100 mM of NaCl to measure K^+^ ion flux (before and after NaCl application) in the root zone by using the MIFE protocol. K^+^ ion flux could be measured 10 mm away from the root tip because fluxes were higher there, and it could be used as a screening tool to measure salinity tolerance in a dose- and time-dependent manner of NaCl, for example, 100 mM of NaCl treatment for 30 min.

In the present study, it was observed that 10 min after adding NaCl, the *OE1* and *OE2* transgenic lines showed a significant decrease in K^+^ ion efflux, indicating an increase in K^+^ ion influx as compared with *Wt*; similarly, *OE5* and *OE7* have shown higher K^+^ ion influx as compared with the fluxes recorded in *Wt* in response to 100 mM of NaCl stress (**Figure 8**). However, relatively more K^+^ ion influx was observed in the *OE1* and *OE2* lines as compared with that in the *OE5* and *OE7* lines (**Figures 9**, 10). Among all *AtCIPK16* overexpressing wheat lines, the *OE2* line exhibited significantly higher and stable K^+^ ion retention in root tissues and demonstrated higher tolerance to salt stress (**Figure 9**). Finally, K^+^ ion efflux gradually recovered in the next 35 min, and a decrease in K^+^ ion efflux was recorded and an increase in K^+^ ion influx was observed in the flux profile of transgenic lines.

**Figure 7:**
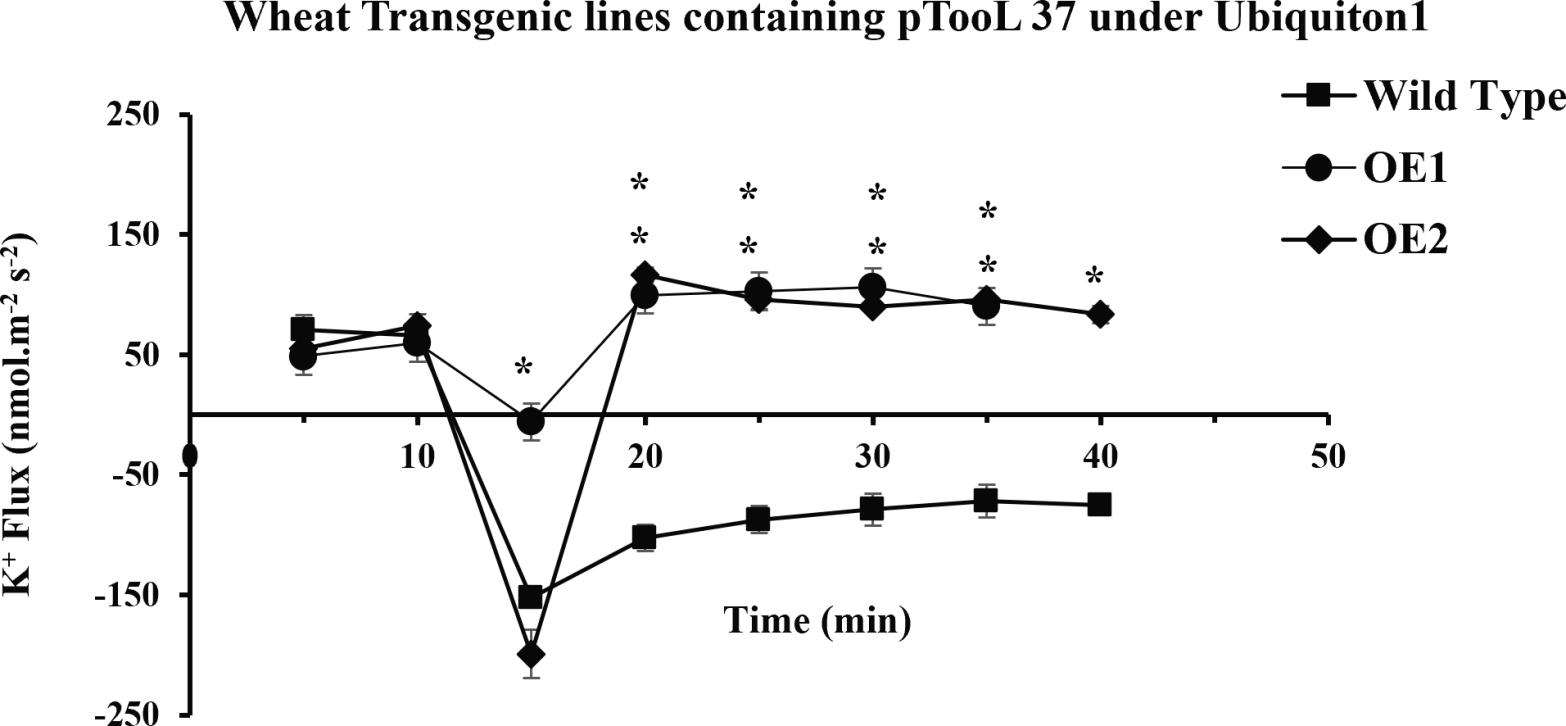
Transient K+ flux responses under the Ubiquitin 1 promoter. K+ flux profile of 5-day-old wheat seedlings along the root axis. The net K+ flux of *OE1, OE2* (pTOOL37 + UBI1 + AtCIPK16), and wild-type wheat cultivar Faisalabad-2008 was measured along the root axis with time and distance (10 mm away from the root tip) before and after 100 mM of NaCl treatment. Average K+ retention ability was found to increase in transgenic lines. Student’s t-test was employed for the significant difference between means at the significant level of *P<0.05 and represented as mean ± standard error (SE). (*) indicates significance; all the rest are non-significant.

**Figure 8:**
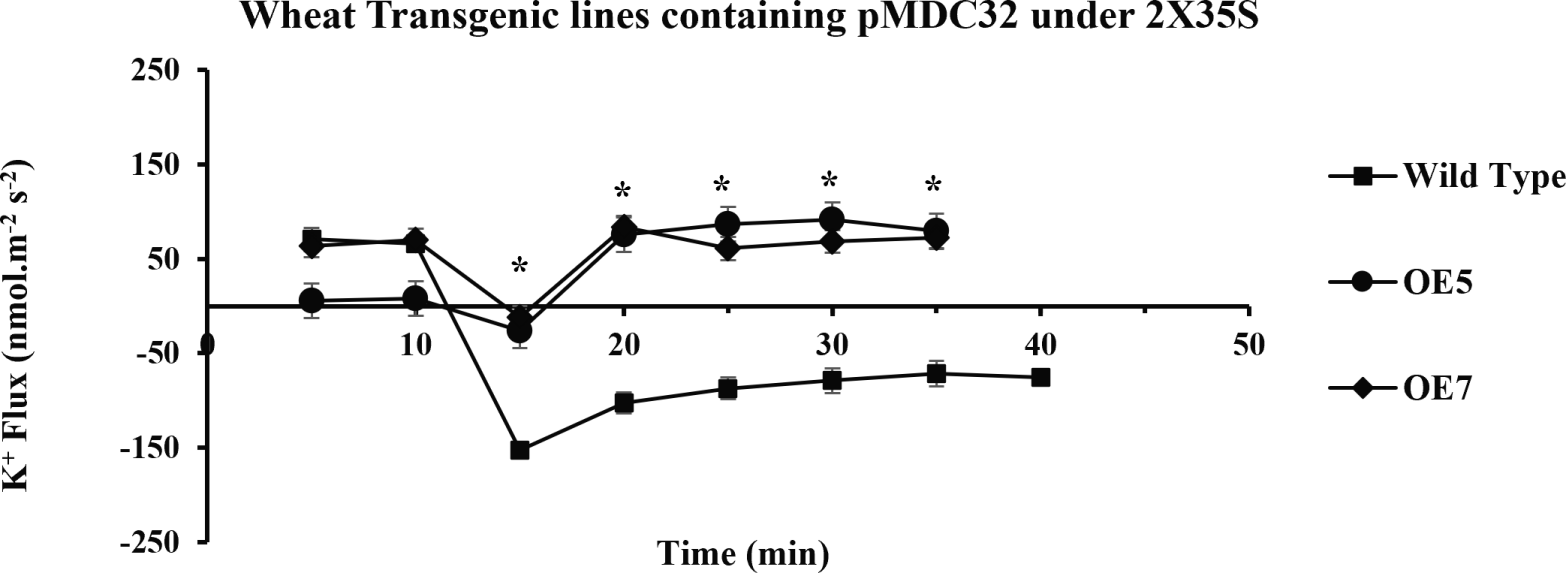
Transient K+ flux responses under the 2XCaMV35S promoter. Flux was measured from the OE5, OE7 (pMDC32 + 2XCaMV35S + AtCIPK16), and wild type before and after 100 mM of NaCl addition, in the mature zone 10 mm away from the root tip. In transgenic wheat lines, a significant increase in K+ influx (retention) was found as compared to that in the wild-type (Wt) wheat cultivar. Student’s t-tests were conducted to evaluate the results and ascertain the significant difference between means at the significant level of *P<0.05 and represented as mean ± standard error (SE).

**Figure 9:**
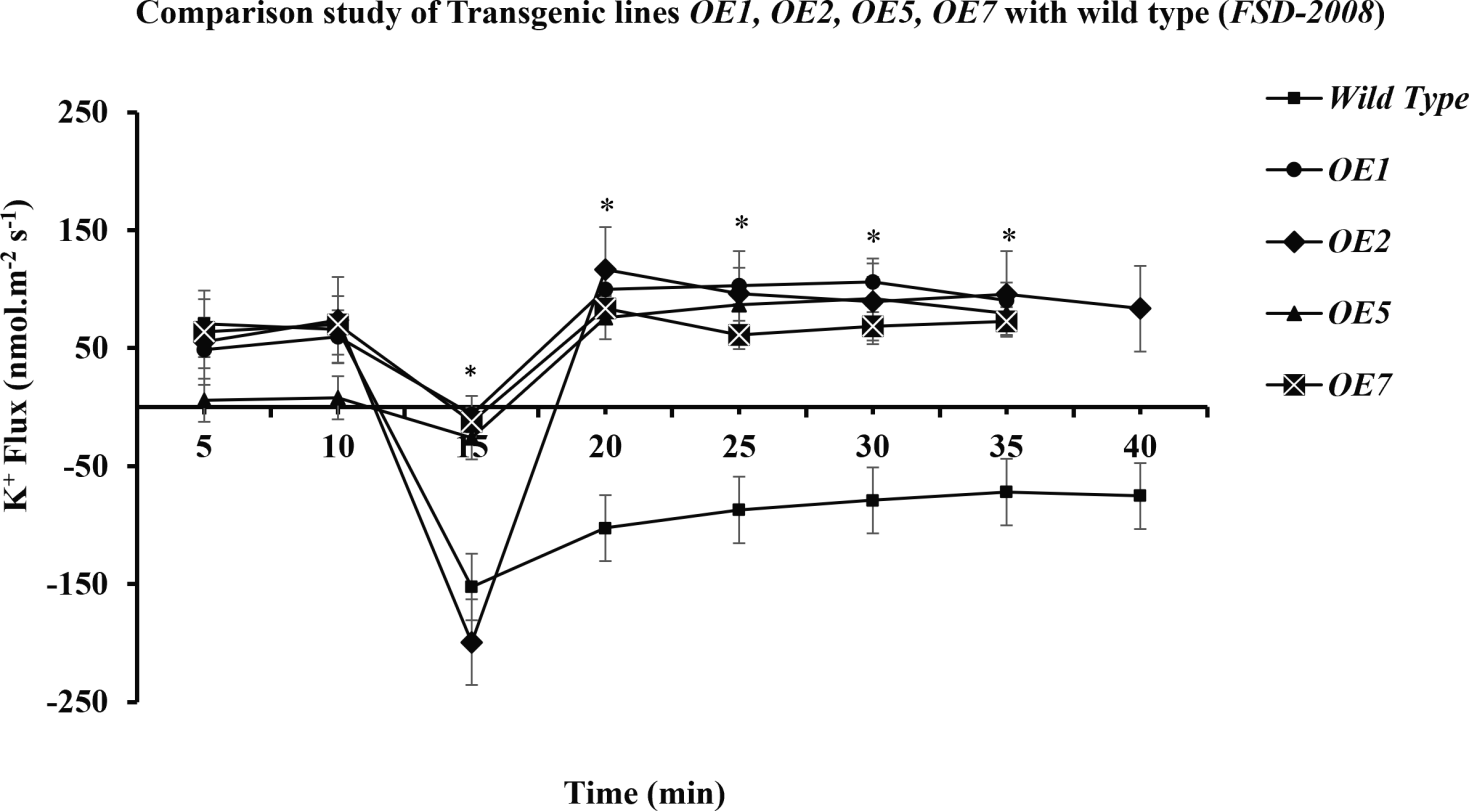
Comparative study of K+ retention in different overexpressed lines and wild type. The performance of transgenic lines under 100 mM of NaCl stress is better in comparison with that of the wild type (Wt) (Fsd-2008), respectively. Among the transgenic wheat lines, *OE2* is performing better than the *OE1, OE5*, and *OE7* transgenic lines. Student’s t-tests were conducted to evaluate the results and ascertain the significant difference between means at the significant level of *P<0.05 and represented as mean ± standard error (SE).

### Variation in NaCl-induced K^+^ flux in transgenic wheat lines

Results have shown that there is a significant difference between the flux profiles of overexpressed and *Wt* wheat lines. Overexpressing wheat lines exhibited a change in flux profile by a significant increase in K^+^ ion influx (a decrease in K^+^ ion efflux) after 10-25 min of salt exposure. K^+^ ion efflux of different magnitudes was measured upon salt exposure in each wheat line. A transient peak showing an increase in K^+^ ion efflux (for 10 min) was observed in response to NaCl application. Six overexpressed wheat lines showed a significant difference in steady-state potassium ion flux profiles after the transient potassium ion efflux peak lasted for 10-20 min. In overexpressed wheat lines, a steady and stable K^+^ ion influx was observed after 20-25 min of salt exposure. A non-significant K^+^ ion efflux was observed in most of the wheat transgenic lines as compared with the *Wt* (a significant K^+^ ion efflux was observed) immediately after exposure to salt stress. In *OE1* (90.2519 nmol m^−2^ s^−1^), *OE2* (95.78772 nmol m^−2^ s^−1^), *OE5* (79.50103 nmol m^−2^ s^−1^), and *OE7* (72.67819 nmol m^−2^ s^−1^), a steady-state K^+^ ion influx was recorded compared with that in the *Wt* (−71.7871 nmol m^−2^ s^−1^) at the same time interval (at 35 min). *Wt* K^+^ ion flux still shows an efflux of potassium, and no K^+^ ion influx was observed at any point in the flux profile under salt stress. A significant (*P* ≤ 0.05*) positive correlation was observed between *AtCIPK16*-induced K^+^ retention and overall salinity tolerance in transgenic wheat lines.

## Discussion

Abiotic stresses can provoke a series of plant responses. Among them, salt stress is considered to have the most adverse effects, affecting ultrastructural cell components and cell metabolism and altering membranous structures and plant physiology. Salts can also cause an ionic imbalance in the cell resulting in ionic toxicity. Specific effects of salt stress on plant metabolism, especially leaf senescence, are associated with the accumulation of toxic Na^+^ and Cl^−^ ions and the depletion of K^+^ and Ca^2+^ ions (Arif et al., 2020; El Sabagh et al., 2021).

Most of the earlier efforts had been made to develop salt tolerance in plants and to investigate the mechanism involved in the exclusion of Na^+^ ions from plants to prevent its transport into the shoot or excessive accumulation in the vacuole. Later, many studies demonstrated no relationship between leaf Na^+^ ion content and salinity tolerance in wheat. So, Na^+^ ion exclusion did not prove to be the single factor associated with salt tolerance in wheat plants. However, Na^+^ ion exclusion and K^+^ ion retention in the root cells may be considered a more reliable strategy for imparting salinity tolerance in wheat as was observed in barley (Wu et al., 2013). Previously, different salinity-tolerant mechanisms at the physiological and molecular levels had been studied extensively. The aim of our present study was to explore research advances with the transgenic approach and genetic biotechnology (Ibrahimova et al., 2021).

In the present study, a locally adapted wheat cultivar was transformed with the *AtCIPK16* gene and evaluated for its salinity tolerance, as it has been reported that *AtCIPK16* has no homolog or ortholog in wheat (*T. aestivum*) and barley (*H. vulgare*) (Amarasinghe et al., 2016). Approximately 1.7% transformation efficiency (TE) was recorded in transgenic wheat using the *AGL-1* strain of *A. tumefaciens*. Although many factors are usually involved in the effective delivery of transgene, the *AGL-1* strain of *A. tumefaciens* was especially employed for its simplicity, rapidity, and well-known hypervirulence in monocot for enhanced transformation efficiency (Li et al., 2021). Previously, Raja et al. (2009) also reported an 8% transformation efficiency by using calli originating from immature wheat embryos in the same cultivar *Faisalabad-2008*. Khan et al. (2015) obtained 5% TE through *AGL-I* by infecting wheat calli. Similarly, Wei et al. (2017) reported 3.70%-5.90% TE by using *AGL-I* for the transformation of the *TaPIMP2* gene under constitutive promoter in wheat crops. Recently, Wu et al. (2018) obtained 0.60-9.70 transformation efficiency by using the same strain of *A. tumefaciens* in wheat.

Furthermore, the expression analysis of the *AtCIPK16* gene under the *UBI1* and *2XCaMV35S* promoters revealed significantly higher gene expression in all transgenic wheat lines at 100 mM of NaCl treatment as compared with the *Wt* (**Figure 5**). The higher transcript level of transformed wheat lines represented presumably efficient Na^+^ ion sequestration into the root vacuoles, restricting the transport of Na^+^ ions into the shoot and leaf tissues. Moreover, the transcripts of the *AtCIPK16* gene under the *UBI1* promoter in transgenic wheat lines exhibited a significantly higher level than the transgenic lines expressing the same gene under the *2XCaMV35S* promoter. Annum et al. (2022) reported the expression of the *AtPLC5* gene at a significantly higher level when expressed under the *UBQ10* promoter upon exposure to heat stress in transgenic wheat. Tao et al. (2015) found that the *UBI1* promoter was significantly active in monocotyledonous plants, especially in cereals (wheat, rice, maize, and barley), and it brought a higher transgene expression level. However, the *CaMV35S* promoter was found to constitutively enhance gene expression in dicots (chickpea, soybean, etc.), but its activity was reduced in monocots (wheat, rice) (Li et al., 2021). In the current study, it was observed that the expression of the *AtCIPK16* gene under the *UBI1* promoter enabled transformed wheat lines to tolerate salinity stress quite effectively. Transgenic wheat lines, i.e., *OE1* and *OE2* expressing *AtCIPK16* under the *UBI1* promoter, were performing better as compared with the *OE5* and *OE7* lines expressing *AtCIPK16* under the *2XcaMV35S* promoter (Figure 10). However, overexpressing *AtCIPK16* transgenic wheat lines showed significantly better growth performance under 100 mM of salinity stress level as compared with the *Wt* (non-transgenes), presumably due to better K^+^ ion retention. To validate this hypothesis, Na^+^/K^+^ ion fluxes were determined using MIFE.

The MIFE technique has offered a significant conceptual development as well as advancement to understand the adaptive responses and cellular membrane transport processes of plants, fungi, bacteria, animal cells, and biofilms related to different environmental stress conditions (Shabala et al., 2012; Buchroithner et al., 2021). Newman (2001), an Australian scientist, invented this technique in 2001 by using ion-selective microelectrodes to determine the non-invasive measurement of ion fluxes (**Figure S5**). This technique was also previously used to study the regulation of ion transport and cyclic nucleotide-mediated transport *via* the root plasma membrane in *A. thaliana* (Ordoñez et al., 2013). It was also reported to be specifically used in wheat plants to determine the K^+^ ion retention ability in plant leaf mesophyll tissues as mandatory components involved in the plant salinity tolerance mechanism (Wu et al., 2015). Salinity tolerance enhanced by cytosolic K^+^ retention in maize roots possessed by the fungus *Piriformospora indica* was also assessed by MIFE (Yun et al., 2018). Wang et al. also found a major QTL responsible for the overall salinity tolerance in cereals (wheat and barley) by using the MIFE technique (Wang et al., 2019). Therefore, in the recent study, the MIFE technique was employed to determine the K^+^/Na^+^ ion fluxes. It was observed that selected transgenic lines used in this study and *Wt* exhibited different flux proportions when exposed to salinity stress of 100 mM of NaCl and showed a significant difference in net K^+^ ion fluxes/retention. Initially, NaCl induced transient potassium (K^+^ ions) efflux upon the addition of 100 mM of NaCl in the root epidermal cells due to excessive salt availability in the rhizospheric region, and then a transient peak of K^+^ ion efflux gradually shifted to the K^+^ ion influx (K^+^ retention) (**Figures 7** **–** **9**). Similarly, K^+^ ion efflux was also observed in wheat and barley plants upon the start of salt stress signaling (Wang et al., 2018). Therefore, the flux profile of the overexpressed lines *OE1* and *OE2* showed significantly higher K^+^ ion influx and Na^+^ ion efflux from the plasma membrane than that of the lines *OE5* and *OE7* and *Wt* upon exposure to 100 mM of NaCl. Zarza et al. (2019) performed a biochemical analysis of PIP_2_-mediated signaling involved in the K^+^ ion flux of plants by using MIFE in *A. thaliana*.

K^+^ is the essential ion required for most of the plant processes and metabolic pathways. Potassium ion fluxes across the plasma membrane are controlled and channeled by fine-tuned ion channels (outward rectifying K^+^ channels). Thus, maintaining the intracellular K^+^ ion level is essentially required, especially in the challenging condition of salt stress for plant survival. Low potassium homeostasis causes K^+^ ion stress which initiated the Ca^2+^ ion signaling pathway by the activation of CIPKs, i.e., *AtCIPK16* interacts with the K^+^ ion channels (AKT1) that maintain the cytosolic Na^+^/K^+^ ion ratio by regulating salt exclusion from the root and shoot tissues under salinity stress to confer salinity tolerance upon overexpression in *A. thaliana* and barley plants. The *AtCIPK16* gene is positively involved in salinity tolerance in *A. thaliana* (Van Kleeff et al., 2018). Similarly, Zhao et al. (2009) reported the involvement of *ZmCIPK16* in high salinity, drought tolerance, and ABA signaling in *Zea mays*. Conversely, CIPK3, CIPK9, CIPK23, and CIPK26 were reported to interact with CBL 2/3 to maintain K^+^ homeostasis in *Arabidopsis* (Tong et al., 2021). However, the CBL–CIPK complex also takes part in the regulation of Mg^+2^, Na^+^, and ${\text{NO}}_{3}^{-}$ ions (Verma et al., 2021). Xiaolin et al. (2022) proposed the involvement of *CqCIPK-CBL* genes in quinoa for improving salinity, cold, and drought tolerance in transgenic and molecular breeding, indicating functional diversity and regulation at the transcriptional level in multiple ways. Similar results were described by Ma et al. (2020) in apple (*Malus domestica*) where *MdCIPK13* was reported to regulate the downstream phosphorylation of the *MdSUT2.2* gene to enhance salinity tolerance (Ma et al., 2020). *AtCIPK23* interacts with *AtCBL1* and *AtCBL9* followed by downstream phosphorylation that activated AKT1 (*Arabidopsis* potassium transporter 1) to maintain potassium homeostasis in the root cells of rice species (Mao et al., 2022). *CDPK13* (calcium-dependent protein kinase 13) had shown a specific role to establish phosphorylation of the guard cell and potassium influx channels, resulting in inward rectification of K^+^ ion channels (KAT1 and KAT2) for the retention of K^+^ ions in *A. thaliana* (Van Kleeff et al., 2018). SOS3 (*CBL4*) is a calcium sensor that receives the sodium-induced rise in cytosolic calcium and interacts with SOS2 (*CIPK24*) to regulate the downstream mechanisms, i.e., phosphorylation, which in turn activates the SOS1 in the plasma membrane to exclude Na^+^ ions from the root cells (Shen et al., 2020). Additionally, Khan et al. (2020) and Wang et al. (2022) proposed the exclusion of Na^+^ ions out of the plasma membrane or Na^+^ sequestration into the vacuole and activation of the antioxidant defense system in plant cells for the generation of salt tolerance. K^+^ ion retention in the cytosol was shown to be essentially required for the enhancement of plant performance under salinity stress (Wang et al., 2018). A positive association between K^+^ ion retention and salinity tolerance was reported in many plant species like wheat, tomato, soybean, and barley. K^+^ ion retention in the plant mesophyll and root tissues was found to be the most important contributing factor in generating salt tolerance (Wang et al., 2019; Khan et al., 2020).

The current study concludes that CIPKs are found to be the key regulators determining pre-transcriptional and post-transcriptional responses to salinity, drought, heat, and cold stresses, and their signaling took part through the membrane system. Published reports have shown the role of the CIPK family in salinity tolerance, but it had not been fully exploited yet, thus strongly suggesting further investigation of this gene family for other abiotic stresses (Shi et al., 2018; Ma et al., 2020). It is also concluded that K^+^ ion retention in the root cells of transgenic wheat lines expressing *AtCIPK16* under the *UBI1* and *2XCaMV35S* promoters showed a high correlation with salinity tolerance in wheat plants. The results were further supported by K^+^ ion influx and retention which was a biochemical indicator for the development of salt-tolerant plants through conventional and transgenic approaches. The results of the present study suggest that K^+^ ion retention in wheat plant root cells is an essential mechanism employed by plants to develop resistance against salinity stress and survival under harsh conditions. Therefore, in addition to emphasizing Na^+^ ion exclusion from the leaf tissues, K^+^ ion retention in the root cells of a plant must be considered while devising a strategy to generate salt tolerance in crop plants.

## Data availability statement

The original contributions presented in the study are included in the article/**Supplementary Material**. Further inquiries can be directed to the corresponding author.

## Author contributions

NAS and MA conceived and planned the experiments. KI designed and conducted the research experiments. MT provided the constructs used in this study. KI and NA evaluated the data on statistical grounds. KI wrote the initial draft. MA and NA reviewed the data and prepared the initial draft of the manuscript. MT and NAS reviewed and edited the manuscript. All authors contributed to the article and approved the submitted version.
