# Supplementary material for: AtCIPK16, a CBL-interacting protein kinase gene, confers salinity tolerance in transgenic wheat
Source: Front Plant Sci. 2023 Mar 16;14:1127311. doi: 10.3389/fpls.2023.1127311 (PMC10060804; doi:10.3389/fpls.2023.1127311)
Supplement: Supplementary file 1 [file DataSheet_1.pdf]

Supplementary material

Figure S1.

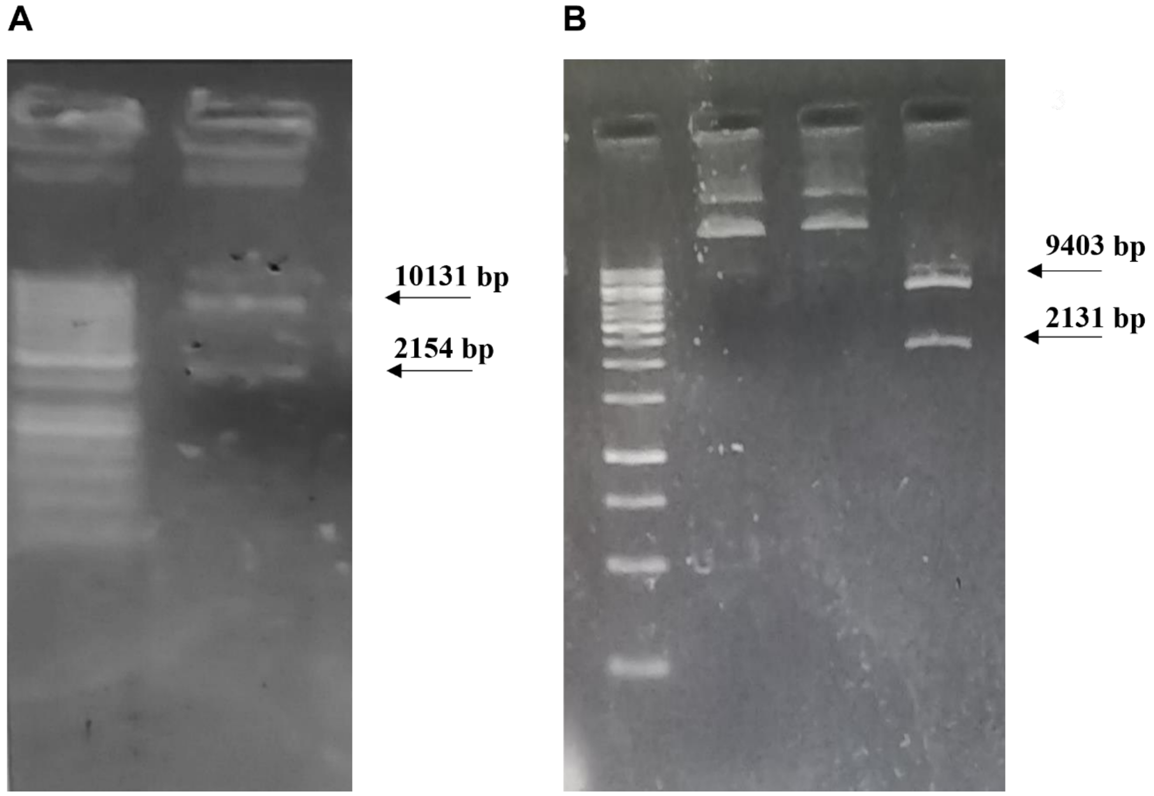

Figure S1. (A) Confirmation of successful cloning of *AtCIPK16* gene under *UBI1*promoter into *pTOOL37* vector. Restriction digestion with *Apa1 and Spe1* restriction enzymes gave two fragments of 10.1 Kb and 2.1 Kb in comparison with 1Kb plus DNA ladder (Fermentas). Lane 1 is 1Kb plus DNA Ladder and Lane 2 is digested clone. **(B)** Confirmation of successful cloning of *AtCIPK16* gene under *2XCaMV35S* promoter into *pMDC32* vector. Restriction digestion with *Sal1 and Spe1* restriction enzymes liberated two fragments of 9.4 Kb and 2.1 Kb in comparison with 1Kb DNA ladder (Fermentas). Lane 1 represents 1Kb DNA Ladder and Lane 2 and 3 is -ve control and Lane 4 represents digested clone.

Figure S2.

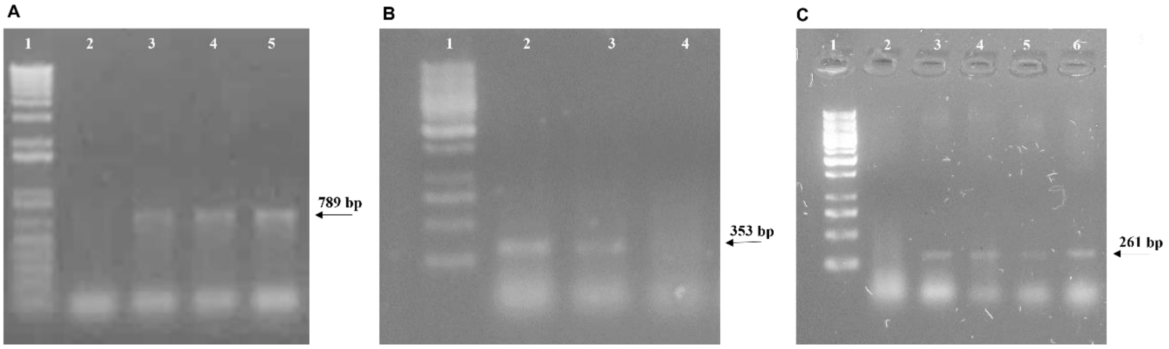

Figure S2. PCR verification of transformation. **(A)** PCR confirmation of *AGL1* strain of *Agrobacterium tumefaciens* transformed with both construct having *AtCIPK16* gene using *Hygromycin* primers as bacterial selection marker that gave band of 789 Kb. Lane 1 is 1Kb plus DNA Ladder and Lane 2 is -ve control and Lanes 3, is +ve control, 4 is transformed culture *of Agrobacterium tumefaciens* of construct 1 and 5 is transformed culture *of Agrobacterium tumefaciens* of construct 2. **(B)** PCR confirmation of transformed *Agrobacterium tumefaciens* strain *AGL1* using promoter *(2X CaMV35S)* and gene (*AtCIPK16*) specific primers that gave band size of 353 bp. Lane 1 is 1Kb DNA Ladder, Lane 2 and 3 are transformed culture of *Agrobacterium tumefaciens of construct 2,* and Lane 4 is -ve control (water). **(C)** PCR confirmation of transformed *Agrobacterium tumefaciens* strain *AGL1* using promoter (*UBI1*) and gene (*AtCIPK16)* specific primers that gave band size of 261bp. Lane 1 is 1 Kb Ladder and Lane 2 is -ve control (water) and Lanes 3 is positive control, 4, 5 and 6 are transformed culture of *Agrobacterium tumefaciens* of construct 1.

Figure S3.

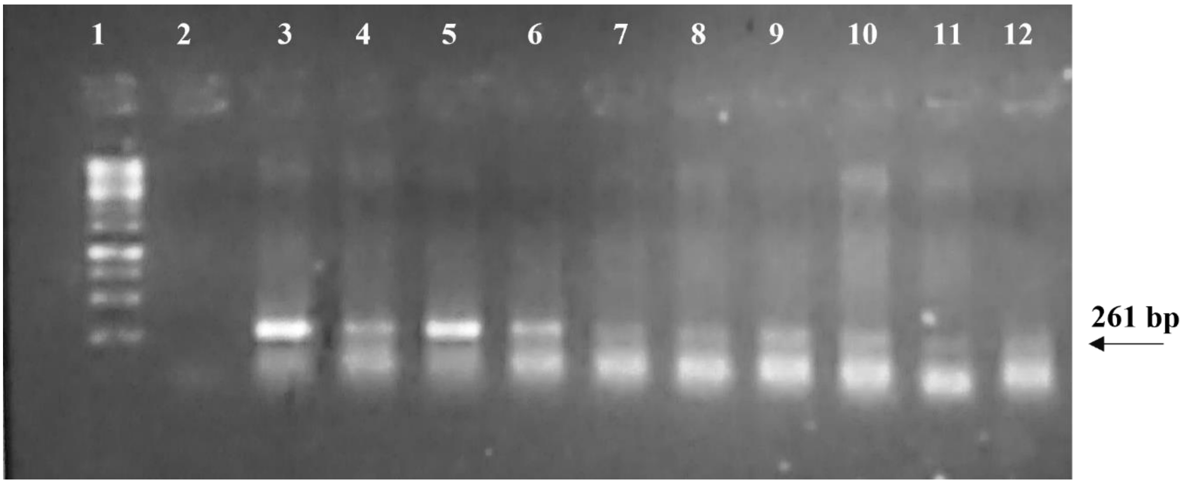

Figure S3. PCR confirmation of the over expressed wheat lines under *Ubiquitin 1. OE1, OE2, OE3* (*pTOOL 37+ UBI1+ AtCIPK16*) under *Ubiquitin1* promoter of size 261bp by gene specific primers. Lane 1 is1Kb DNA Ladder (Fermentas) lane 2 - ve, lane 3 + ve, lanes 4, 5, 6, 7, 8, 9, 10, 11 and 12 are transgenic lines.

Figure S4.

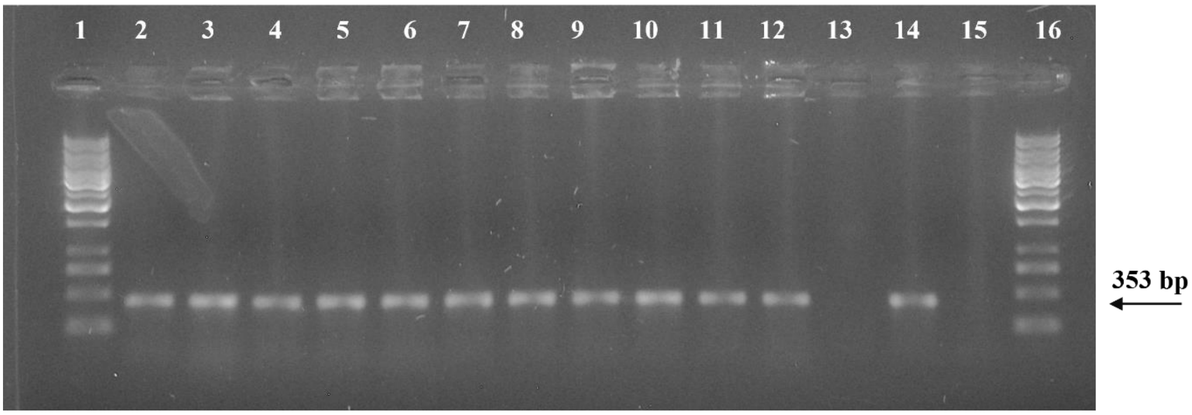

Figure S4. PCR confirmation of the over expressed wheat lines under *2XCaMV35S. OE5, OE6, OE7* (*pMDC32 + 2XCaMV35S + AtCIPK16*) under Cauliflower mosaic virus (*2x CaMV35S* of size 353bp by gene specific primers. Lane 1 is 1 Kb DNA Ladder (Fermentas) lane 2, 3, 4, 5, 6, 7, 8, 9,10,11 and 12 are transgenic lines, lane 13 is -ve, lane 14 is +ve, 15 is -ve and 16 is DNA ladder.

Figure S5.

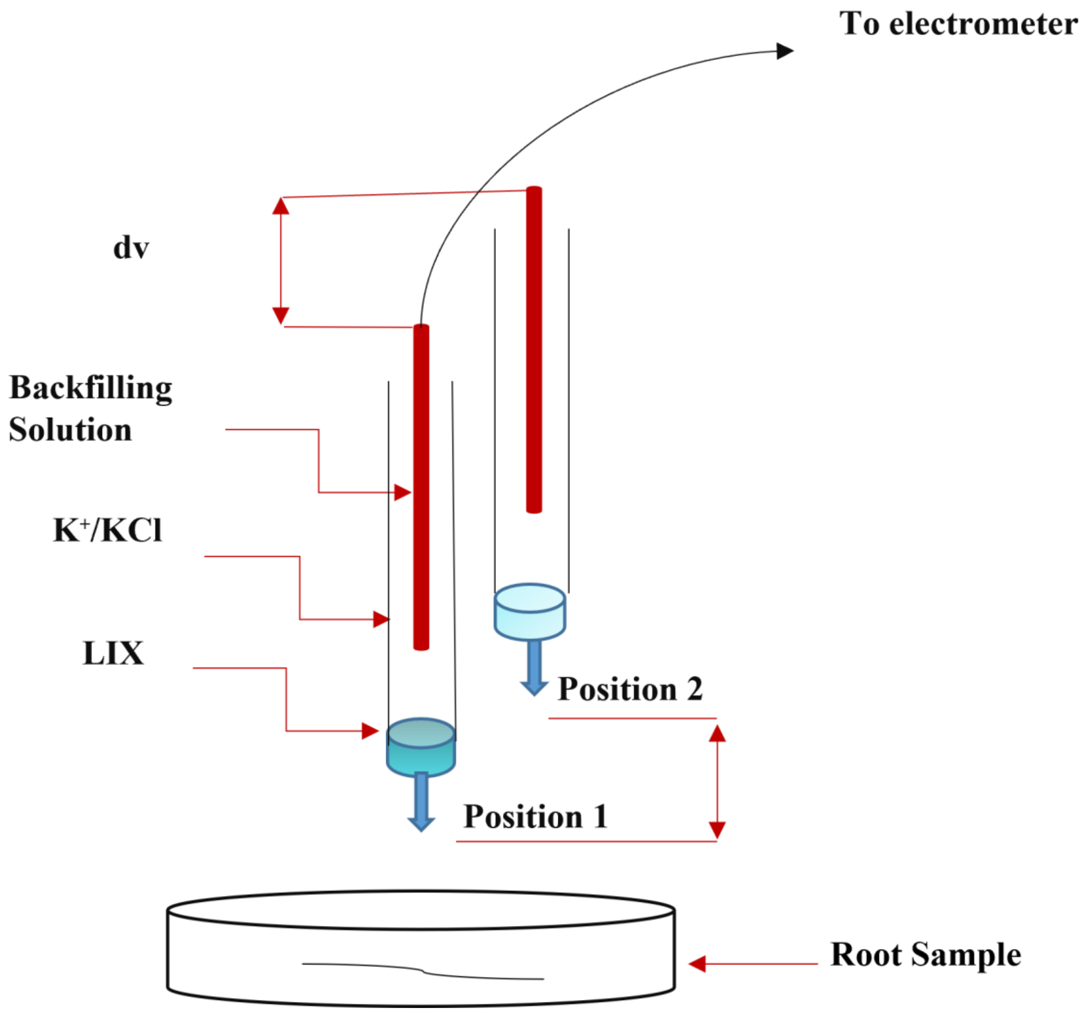

Figure S5. Vibrating microelectrode configuration representation, measuring the K^+ ion fluxes by changing position and movement.

S7

Microelectrode Preparation

Capillary glass tube was used to prepare K^+ LIX tube (GC-150-10; Harvard Apparatus, Kent, U.K), tip was pulled with electrode puller (L/M-3P-A, List Medical Electronics, Darmstadt, Germany) and salinized with tributylchlolorosilane (Fluka 90974). Salinized electrode was immobilized in microelectrode filling station and tip was broken to achieve diameter 2-3µm. In the filling station under microscope, the K^+ LIX tube was immobilized horizontally against blank microelectrode. K^+ backfilling solution was utilized to back-fill the electrode whereas, front was filled by contacting the electrode with the open tip of LIX-containing tube. Prepared microelectrode was stored in the BSM (basic salt medium). Three calibration solutions with different K^+ concentrations (over expected K^+ concentration) in the measuring solution were used to calibrate K^+ microelectrode. 1000, 5000, 10000 µm calibration solutions, which must be higher in range than the K^+ in the BSM. Then AVC file would be created through CHART software automatically using required intercept parameters, correlation coefficients (must be above 0.999) and slope (above 50 mV) of K^+ microelectrode to be displayed.
